# A Global Assessment of the Transcription-Dependent Single Nucleotide Variants Relies on the Characteristics of RNA-Sequencing Technologies

**DOI:** 10.3390/biom16020211

**Published:** 2026-01-29

**Authors:** Xia Zhang, Jiawei Liu, Yabing Zhu, Guixue Hou, Mingzhou Bai, Yuxin Li, Wenbo Cui, Siqi Liu

**Affiliations:** 1 College of Life Science, University of Chinese Academy of Sciences, Beijing 100049, China; zhangxia18@mails.ucas.ac.cn (X.Z.); liyuxin@genomics.cn (Y.L.); cuiwenbo@genomics.cn (W.C.); 2 Department of Proteomics, BGI Genomics, Shenzhen 518083, China; liujiawei1@genomics.cn (J.L.); zhuyabing@genomics.cn (Y.Z.); houguixue@genomics.cn (G.H.); baimingzhou@genomics.cn (M.B.); 3 Department of Biotechnology and Biomedicine, Technical University of Denmark, 2800 Lyngby, Denmark

**Keywords:** RNA-seq, SRS, LRS, g-tSNV, e-tSNV, TSCS, machine learning

## Abstract

Single nucleotide variants (SNVs) are crucial in cancer occurrence and development. SNVs at the transcriptomic level generally come from genomic variants (g-tSNVs) and RNA editing (e-tSNVs). The types and quantities of e-tSNVs remain a subject of debate due to a relatively poor understanding of RNA editing processes. Herein, we developed TSCS (Transcript SNVs Classifier relying on complementary sequencings), a machine learning classifier that integrates short-read (MGI) and long-read (PacBio) RNA-seq data to accurately distinguish true transcript SNVs using stringent criteria. Applied to five colorectal cancer cell lines (HCT15, LoVo, SW480, SW620, and HCT116), TSCS demonstrated superior accuracy and sensitivity, outperforming established tools (GATK, BCFtools, Longshot, RED_ML). It increased the total detected transcript SNVs by 31.83% on average, with g-tSNVs and e-tSNVs exceeding conventional methods by >1-fold and >2-fold, respectively. TSCS achieved mean recall rates of 75.3% for g-tSNVs and 77.2% for e-tSNVs. Notably, for the first time, e-tSNVs were found in a relatively large proportion of total transcript SNVs in cancer cell lines, approximately 40%. Of the identified e-tSNVs, 80% were attributed to the known RNA editing, but the other e-tSNVs did not fall into any known category. Importantly, the e-tSNVs uniquely detected in this study showed distinct patterns in SNV types and genomic locations. Additionally, the transcript SNVs called by TSCS were partially confirmed using experimental approaches, such as Sanger sequencing, RNC-seq, and mass spectrometry. This study lays the foundation for surveying and appraising the cancer-related e-tSNVs.

## 1. Introduction

Cancer is a genomic disorder driven by extensive mutational events [1]. The Cancer Genome Atlas (TCGA) has revealed that approximately 95% of these mutations are single nucleotide variations (SNVs), among which about 91% are nonsynonymous (nsSNVs) that contribute to functional aberrations in cancer cells [2,3,4,5]. Advances in next-generation sequencing (NGS) have led to the identification of millions of nsSNVs, with around 6 million cataloged in the COSMIC database [6]. Concurrently, RNA-seq enables the quantitative detection of transcript-level SNVs, which fall into two categories: those originating from genomic mutations (g-tSNVs), and those arising from RNA editing (e-tSNVs), which are also called transcript SNVs caused by RNA-DNA differences (RDDs) or RNA editing sites [7]. As e-tSNVs are not genetically inherited and can alter amino acid sequences—potentially distorting protein function—they offer a valuable perspective for investigating cancer mechanisms [8,9,10].

Currently, e-tSNVs are primarily identified by excluding g-tSNVs from total transcript SNVs. Databases such as REDIdb, DARNED, RADAR, and REDIportal compile RNA editing events [11,12,13,14]. A key feature of e-tSNVs is their variant allele frequency (VAF), calculated as the ratio of the main variant supporting reads to the total reads at a given locus. This characterizes the allelic representation of detected variants, which is often low, with adenosine-to-inosine (A-to-I) changes in non-coding regions—especially in 3′UTR Alu repeats—being the most common [15]. However, the abundance and diversity of RNA editing events remain controversial [16]. Some studies report widespread editing involving diverse mechanisms [17,18], whereas others highlight high false-positive rates in e-tSNV identification [19,20,21,22]. A major challenge lies in accurately detecting e-tSNVs, particularly those with low VAF. Issues such as transcript strand specificity, splicing complexity, and highly variable expression levels complicate SNV calling in RNA-seq [23,24]. Current transcript SNV callers (e.g., SNVQ [25], SNPiR [23], eSNV-detect [26], MuTect2, and VarScan2 [27],) typically employ a two-stage approach: initial calling using genomic tools like GATK [28] or BCFtools [29], followed by transcript-aware filtering. However, this strategy has key limitations. Genomic callers often discard true low-VAF variants due to poor adaptation to RNA expression characteristics. Moreover, inconsistent filtering criteria across tools lead to low reproducibility, especially for e-tSNVs. To address this, specialized tools such as RNAEditor [30], SPRINT [31], REDItools2 [32], JACUSA2 [33], RDDpred, RED-ML [34], and DeepRed [35,36] incorporate RNA editing features to improve accuracy. However, their reliance on known editing databases limits the discovery of novel events. A robust and comprehensive pipeline for e-tSNV detection is urgently needed.

Accurate e-tSNV identification requires optimized sequencing strategies and analytical methods. Short-read sequencing (e.g., MGI’s DNB-based NGS technology) offers high depth and coverage at a low cost but is prone to mismapping and artifacts, particularly for transcript SNVs [37,38,39,40]. Long-read technologies (e.g., PacBio single-molecular real-time sequencing) yield accurate long reads that improve variant detection but suffer from lower depth in transcriptomic applications [41,42]. Combining both sequencing technologies could leverage their respective strengths. Second, current algorithms often apply arbitrary thresholds that overlook transcript-specific attributes. A quality-focused approach, emphasizing sequencing reliability over preset filters, may improve SNV calling. Third, systematic integrated multi-omics profiling and orthogonal technical validation are essential to distinguish g-tSNVs from e-tSNVs and confirm transcript SNVs. All the considerations above prompted the development of a pipeline to distinguish g-tSNVs and e-tSNVs in cancer cell lines.

In this communication, five cell lines of colorectal cancer (CRC)—HCT15, LoVo, SW480, SW620, and HCT116—were cultured and parallel genomic and transcriptomic sequencing was performed using both MGI and PacBio technologies. With the integration of data advantages from the two sequencings, a BCFtools-based variant calling workflow incorporating machine learning was established to distinguish true SNVs. Predictions were verified via Sanger sequencing, RNC-seq, and mass spectrometry. The systematic investigation to transcript SNVs in different CRC cell lines lays the foundation for overall survey of g-tSNVs and e-tSNVs, and profits discovery of cancer-related e-tSNVs.

## 2. Material and Methods

### 2.1. Cell Culturing for Cell Lines

Five human colorectal cancer cell lines—HCT15 (ATCC^®^ CCL-225™), LoVo (ATCC^®^ CCL-229™), SW480 (ATCC^®^ CCL-228™), SW620 (ATCC^®^ CCL-227™), and HCT116 (ATCC^®^ CCL-247™)—were obtained from the American Type Culture Collection (ATCC, Manassas, VA, USA). HCT15 was maintained in Roswell Park Memorial Institute 1640 (RPMI-1640) medium, while the others were cultured in Dulbecco’s Modified Eagle’s Medium (DMEM). All media were supplemented with 10% fetal bovine serum and 1% penicillin/streptomycin, and cells were incubated at 37 °C in a 5% CO_2_ atmosphere. To ensure robust omics profiling, biological triplicates for each line were established, pooled to minimize culture-specific variation, and subdivided into technical replicates for genomic and transcriptomic sequencing. Independent cell batches were cultivated for all validation studies, including Sanger sequencing of cDNA to verify transcriptional variants, alongside translatomic and proteomic analyses.

### 2.2. Extraction of Genomic DNA, RNA, RNCs, and Proteins from Cell Lines

Genomic DNA was extracted from all five cell lines using a phenol–chloroform–isoamyl alcohol protocol. Cell pellets were lysed in TritonX-100/SDS buffer (Sigma-Aldrich, St. Louis, MO, USA), followed by extraction with glass bead-assisted phenol–chloroform. After centrifugation, the aqueous phase was treated with chloroform–isoamyl alcohol, RNase, and ethanol-precipitated using sodium acetate. DNA was resuspended in a TE buffer and stored at −80 °C.

Total RNA was isolated using TRIzol™ Reagent (Thermo Fisher Scientific, Waltham, MA, USA). Cell lysates in TRIzol were chloroform-extracted, and RNA was precipitated with isopropanol, washed with ethanol, and assessed for quality using an Agilent Bioanalyzer 2100 (Agilent Technologies, Santa Clara, CA, USA).

RNCs were isolated from HCT116 cells using a cycloheximide-based method [43]. Cells were lysed in Triton X-100 buffer (Sigma-Aldrich, St. Louis, MO, USA), and the supernatant was ultracentrifuged through a sucrose cushion. RNC-associated RNA was extracted with TRIzol and quality-checked via Bioanalyzer (Agilent Technologies, Santa Clara, CA, USA).

Proteins were extracted using a urea/thiourea lysis buffer. Lysates were sonicated, reduced with DTT, alkylated with iodoacetamide, and digested overnight with trypsin via FASP membrane purification.

### 2.3. Genomic DNA Sequencing

Genomic DNA was fragmented using a Covaris E220 (Covaris, Woburn, MA, USA), and fragments of 200–400 bp were selected with MGIEasy DNA Clean Beads (MGI Tech Co., Ltd., Shenzhen, China). Library preparation, including end repair, A-tailing, and adapter ligation, was performed using the MGIEasy Universal DNA Library Prep Kit (MGI Tech Co., Ltd., Shenzhen, China). Adapter-ligated DNA was amplified, circularized with the MGIEasy Circularization Kit (MGI Tech Co., Ltd., Shenzhen, China), and sequenced on the DNBSEQ-T7 platform (MGI Tech Co., Ltd., Shenzhen, China) with 150 bp paired-end reads.

### 2.4. RNA and RNC Sequencing

For MGI sequencing, RNA and RNC samples were reverse-transcribed using SuperScript™ II with oligo(dT) priming. cDNA libraries were constructed using the MGIEasy RNA Library Prep Kit and sequenced on the DNBSEQ-T7 (150 bp PE).

For PacBio sequencing, poly(A) RNA was selected, tailed, and reverse-transcribed using SuperScript™ II Reverse Transcriptase and an RT primer (5′-AAGCAGTGGTATCAACGCAGAGTACNNNNNNNNTTTTTTTTTTTTTTTTTTTTTTTTTTTTTTVN-3′). cDNA was amplified, ligated into SMRTbell templates, and sequenced on the PacBio Sequel II system with SMRT Cell 8M. CCS reads were generated under a minimum of three full passes and 90% accuracy.

### 2.5. Proteomics Analyzed Using LC-MS/MS

Digested peptides were fractionated using a C18 column under high-pH conditions, redissolved in 0.1% formic acid, and analyzed on a nanoElute UPLC system coupled to a timsTOF Pro2 mass spectrometer. Peptides were eluted with an acetonitrile gradient (2–80% B) over 60 min at 300 nL/min and 50 °C. MS/MS data were acquired in the data-dependent acquisition (DDA) mode.

### 2.6. DNA Sequencing Bioinformatics

Raw DNA sequencing data were filtered with SOAPnuke (v1.5.6) to remove adapters and low-quality reads. Clean reads were aligned to the GRCh38 reference genome using BWA. Post-alignment processing was performed using Picard tools: SortSam, AddOrReplaceReadGroups, MarkDuplicates, and ReorderSam. Variant calling was conducted using GATK (v4.2.2.0) with BaseRecalibrator, ApplyBQSR, and HaplotypeCaller (-ERC GVCF). Subsequent steps included VariantRecalibrator, ApplyVQSR, and hard filtering (AB < 0.2||MQ0 > 50). Final variants were annotated using ANNOVAR (version 2020-06-08).

### 2.7. RNA and RNC Sequencing Bioinformatics

MGI-derived RNA and RNC reads were preprocessed with SOAPnuke and aligned to the reference genome via STAR (v2.7.10b) using the two-pass mode with transcriptome quantification. GATK (v4.1.7.0) and BCFtools (v1.13) were used for transcript SNV callings. To ensure a strictly comparable and fair evaluation, GATK and BCFtools were applied to the same pre-processed BAM files in parallel. The pre-processing includes initial quality control, alignment, and SplitNCigarReads. For filtering the GATK calls, the quality filtering consisted of Variant Quality Score Recalibration (VQSR), hard-filtering (QD < 2.0, FS > 60.0, SOR > 3.0, etc.), and the selection of high-confidence SNPs. For filtering the BCFtools calls, quality filtering options built into the software were used with the option at (-s LOWQUAL -e ‘QUAL < 10||FMT/DP < 5’ --SnpGap 5) to remove low-quality sites, such as QUAL < 10, DP < 5, or variants within 5 bp of an indel. The parameters of GATK and BCFtools in pre-processing and calling are enclosed in File S1 “Supplementary Parameters”. RED-ML identified e-tSNVs using dbSNP138, simpleRepeat, and Alu databases. Transcript abundance was estimated with StringTie and normalized using FPKM (≥1 threshold).

PacBio FLNC reads, generated via SMRT Link and lima, were aligned with GMAP. GATK, BCFtools, and Longshot were used for transcript SNV calling; RED-ML was applied for e-tSNV detection.

RNA-seq libraries were prepared using a non-strand-specific protocol. To mitigate potential misalignment artifacts, the generally accepted protocols were implemented: (1) with exon annotation to map variant candidates against the GENCODE v38 (GRCh38.p13) annotation; (2) with strand assignment to locate variants within exons and the transcriptional strand orientation (“+” or “−”) of the corresponding gene; and (3) with a biological base change to convert the raw “alignment-based base change” (as observed in the sequencing data) to the corresponding “biological base change” on the sense strand.

### 2.8. Proteomic Bioinformatics

Peptides were identified using MaxQuant (v2.0.1.0) against the UniProtKB/Swiss-Prot database (Oct 2020 release) with trypsin digestion, two missed cleavages allowed, carbamidomethylation (fixed), oxidation and acetylation (variable), and FDR ≤ 0.01. For SAV detection, a custom library including reference and variant sequences was searched similarly. Identification quality was assessed using posterior error probability (PEP).

### 2.9. Verification of Transcript SNVs Using Sanger Sequencing

Genomic regions containing target SNVs were amplified from DNA and cDNA using primers (Supplementary Table S1). PCR products were gel-purified, cloned into pClone007, and transformed into *E. coli*. Positive clones were validated using PCR and sequenced (GENEWIZ). Data were analyzed using Sequencing Analysis 5.2 and MEGA6.

### 2.10. Analysis of SNV Characteristics

SNV analyses were performed using Python (v3.9.13). Sequencing read depths in exons were estimated from BCFtools-derived genotype likelihoods and RefSeq hg38 annotations. SNV features (VAF, type, genomic region) were extracted from VCF files and ANNOVAR annotations. Mutated peptides were identified via MaxQuant using a custom UniProt-SAV library. Protein isoelectric points (pI) were computed using ProtParam (Expasy, https://web.expasy.org/protparam/, accessed on 25 January 2026), and functional predictions were conducted using enrichGO in R (v4.2.0).

### 2.11. Model Construction for Transcription-Dependent SNV Calling

An SNV library comprising true and false variants from BCFtools calls (MGI and PacBio) was constructed. Datasets were split into training (80%) and testing sets. Five algorithms—RF, SVM, LR, XGB, and GBDT—were evaluated over 100 iterations based on the selected features. A total of 12 features were extracted directly from the VCF files and were primarily used for the assessment with confidence, alignment quality, and potential systematic biases at individual variant sites. Besides, a novel set of nine features, which were derived from the overall sequence and variant-calling context of the exon harboring, was introduced to enhance the model’s ability, such as exon_RSD, exon_SNV_count, and exon_rank_top. The best performing model was selected based on AUROC.

### 2.12. General Data Analysis

The correlations of SNV VAFs between MGI and PacBio were estimated using Spearman’s correlation analysis with the spearmanr module of scipy.stats in Python. All the plots for figure generation were based on matplotlib.pyplot packages in Python and ggplot2 package in R.

## 3. Results

### 3.1. Discriminator Establishment Towards Transcript SNVs Based on Sequencing Data from MGI and PacBio

**Figure 1:**
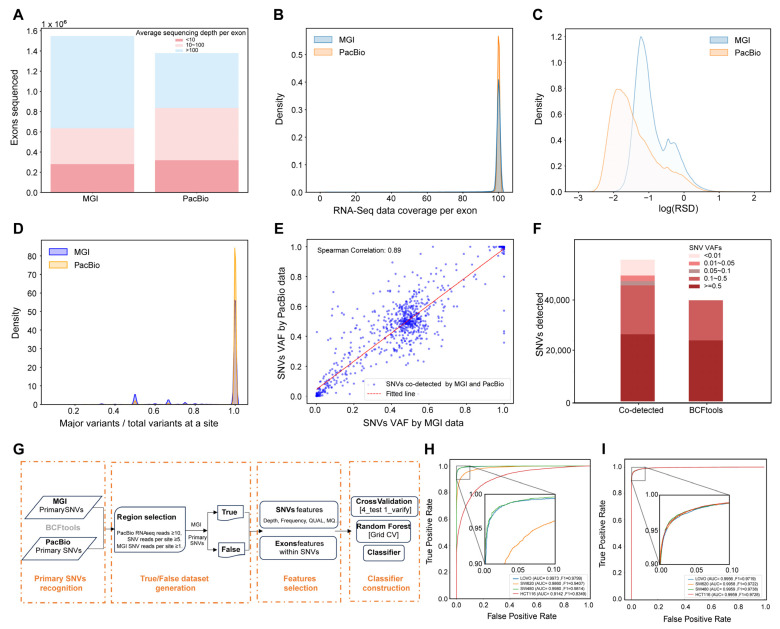
Construction of the workflow to discover transcript SNVs in cancer cell lines. (**A**) Comparison of the identified exons between two sequencing datasets according to the sequencing depth per exon in HCT15. (**B**) Comparison of the sequencing coverage per exon between two sequencing datasets in HCT15. (**C**) RSD distribution of the sequencing depth per exon in the two sequencing datasets in HCT15. (**D**) Evaluation of data consistency for SNVs identified by two different sequencing approaches in HCT15. (**E**) Correlation of VAFs for the SNVs co-identified by two different sequencing approaches using the Spearman correlation analysis in HCT15. (**F**) Comparison of the SNVs extracted by the two different calling approaches according to the SNVs at different levels of VAF in HCT15. (**G**) The infrastructure of the transcript SNVs callings (TSCS) workflow developed in this study. (**H**) Based on the TSCS constructed using the HCT15 sequencing data, the transcript SNVs in LoVo, SW480, SW620, and HCT116 were predicted. (**I**) Based on the TSCS models constructed using the LoVo, SW480, SW620, and HCT116 sequencing data, the transcript SNVs in HCT15 were predicted in different discriminators.

We systematically evaluated the performance of MGI and PacBio platforms in transcriptomic SNV calling across five colorectal cancer cell lines: (1) The identified exons and average of sequencing depth per exon derived from the two sequencing technologies in the same cell line were compared, as exemplified by HCT15 (Figure 1A). MGI identified more exons with a high sequencing depth (59%) than PacBio (39%), demonstrating its superior exon coverage. This pattern was uniformly observed in all five cell lines (Figure S1A); (2) Specifically looking at the RNA-seq data coverage in an exon, PacBio exhibited higher densities of the RNA-seq data with complete coverage than these found by MGI, as depicted in Figure 1B and Figure S1B, suggesting that PacBio with longer reads can provide high-quality data for fully covering entire genes; (3) The relative standard deviation (RSD) of sequencing depth per exon was introduced to describe the uniformity of RNA-seq data in an exon: the higher the RSD values, the stronger the flexibilities of sequencing data within exons. Figure 1C and Figure S1C display the density distribution of RSD generated from either MGI or PacBio sequencing and indicate that the peak of the RSD density curve from PacBio is lower than that from MGI. Thus, the uniformity of the PacBio sequencing data is reasoned over that of MGI; and (4) Primary SNV calling with BCFtools consistently yielded a greater number of raw variants from the MGI data compared with PacBio across all five cell lines (e.g., HCT15: 22,502,174 vs. 3,503,950; for complete counts see Table S1). The consistency of primary SNVs at a site (CPS) is defined as the ratio of the main variant reads divided by the total variants reads at a site, and the CPS profiles of MGI and PacBio are presented in Figure 1D and Figure S1D, implicating that the average of CPS with 100% consistency in PacBio is significantly higher than that in MGI (e.g., HCT15: 96% vs. 83%). Even though the total primary SNVs called from PacBio are much less than MGI, the data quality of the SNVs calling is highly acceptable in PacBio. Selecting SNVs with a read depth ≥ 5 and 100% consistency yielded 55,083 variants co-detected by both technologies in HCT15, showing strong VAF correlation (Spearman r = 0.89; Figure 1E). The VAFs of these co-detected SNVs, 55,083 SNVs, are broadly divided into five groups indicated in Figure 1F. Importantly, 15,294 SNVs with lower VAF groups (<0.1) are totally removed by the filtration in BCFtools. This pattern—high concordance among co-detected variants coupled with the systematic loss of low-VAF SNVs—was consistently observed across all additional cell lines (Figure S1E,F). It is clear that BCFtools that simply set transcriptional features as filtration criteria result in mis-calling of the SNVs with low VAFs. Collectively, our analyses demonstrate that PacBio (LRS) provides more uniform and complete sequencing data for reliable SNV calling, whereas MGI (SRS) offers greater depth for variant discovery. To ascertain whether these differences were technology-inherent rather than batch-specific, we compared additional SRS and LRS datasets from the same HCT15 cell line generated across independent laboratory batches. As shown in Figure S1G, the disparities in exon coverage, sequence read distributions, and primary SNV calls between SRS and LRS were substantially greater than those observed between different SRS batches. This confirms that the observed distinctions are systematic attributes of the sequencing technologies. Therefore, we conclude that the complementary strengths of SRS and LRS can be leveraged to enhance alignment accuracy and improve SNV detection performance when integrated.

To leverage the complementary strengths of both sequencing technologies, we developed the Transcript SNVs Classifier based on Complementary Sequencings (TSCS) pipeline (Figure 1G), which integrates four technical phases: (1) selection of SNV candidates: collecting all the genomic regions with high confidence upon PacBio data (reads ≥ 10) and looking for possible SNVs in the regions using BCFtools that are supported by reads ≥ 5 per site; (2) definition of true and false SNVs: the MGI-called SNVs that overlap with the PacBio SNV candidates are defined as true SNVs; conversely, the MGI-called SNVs located in genomic regions where PacBio achieves ≥100× coverage but are not called by PacBio are defined as false SNVs; (3) extraction of discriminative features: discriminative features gained from VCF files and exon uniformity metrics (Figure S2); and (4) construction of the machine learning model: based on datasets with true and false SNVs and discriminative features, SNVs classifiers are developed and evaluated using five algorithms and the algorithm with the best performance is selected as the final classifier.

The discriminator performance for the identification of true SNVs was examined using two approaches. First, using RNA-seq data from five cell lines (HCT15, LoVo, SW480, SW620, and HCT116), we trained a Random Forest model on HCT15-derived true and false SNVs as mentioned above and tested it on the other four cell lines. The model achieved an AUC of 0.91 across all test sets (Figure 1H), demonstrating robust cross-cell-line recognition of true and false SNVs. Second, we trained four independent discriminators on SNVs from LoVo, SW480, SW620, and HCT116, respectively, then applied each to classify HCT15 SNVs. All four discriminators successfully identified HCT15 SNVs with AUC values exceeding 0.99 (Figure 1I), and showed 95–98% overlap in their true SNV predictions, indicating high reproducibility. These results establish that combining MGI and PacBio data to train a discrimination model seems workable as a universal strategy to find out the reliable signals of transcript SNVs, at least in cell lines.

Furthermore, TSCS demonstrated a consistent performance across sequencing platforms and experimental batches. Evaluation of the PacBio and MGI data from the same cell lines revealed that over 89% of SNVs identified from PacBio were replicated in MGI datasets, with similar concordance rates observed across all cell lines (89–94%, Table S2). To further assess reproducibility, we applied TSCS to replicate SRS datasets from HCT15 generated across different experimental batches. The results showed 85% (50,755/59,644) concordance in SNV calls within commonly covered regions. These results establish TSCS as a robust method for cross-platform and cross-batch SNV analysis, providing highly reproducible results regardless of sequencing technology or experimental conditions.

### 3.2. Performance Comparison of Transcript SNVs Callings Among Different Software

To evaluate the performance of transcript SNV calling across different software, we cultured five cell lines (HCT15, HCT116, LoVo, SW480, and SW620) and performed parallel genomic sequencing and RNA-seq. Genomic SNVs were called using GATK, while primary transcript SNVs were identified using GATK and BCFtools for both MGI and PacBio data, with Longshot [44] additionally applied to the PacBio data. We focused on comparing transcript SNVs called by TSCS against those from other tools. Following strict filtering based on transcriptional parameters and sequencing data quality, the final SNV sets were categorized into g-tSNVs that are shared by genomic and transcriptomic SNVs that were originated from genomic mutations and e-tSNVs that are uniquely found in transcriptomic SNVs that were mostly generated from the RNA editing process. For the e-tSNV comparison, RED-ML was also implemented.

Whether the e-tSNVs identified by TSCS followed the well-accepted principle in this area was assessed according to three criteria: (1) if A-to-G (corresponding to A-to-I editing) is largely enriched; (2) if 3′ untranslated regions (3′UTRs) of genes are concentrated; and (3) if low frequencies of the variant are predominant. Our results show that the general characterization of e-tSNVs is fully demonstrated in all the five cell lines.

**Figure 2:**
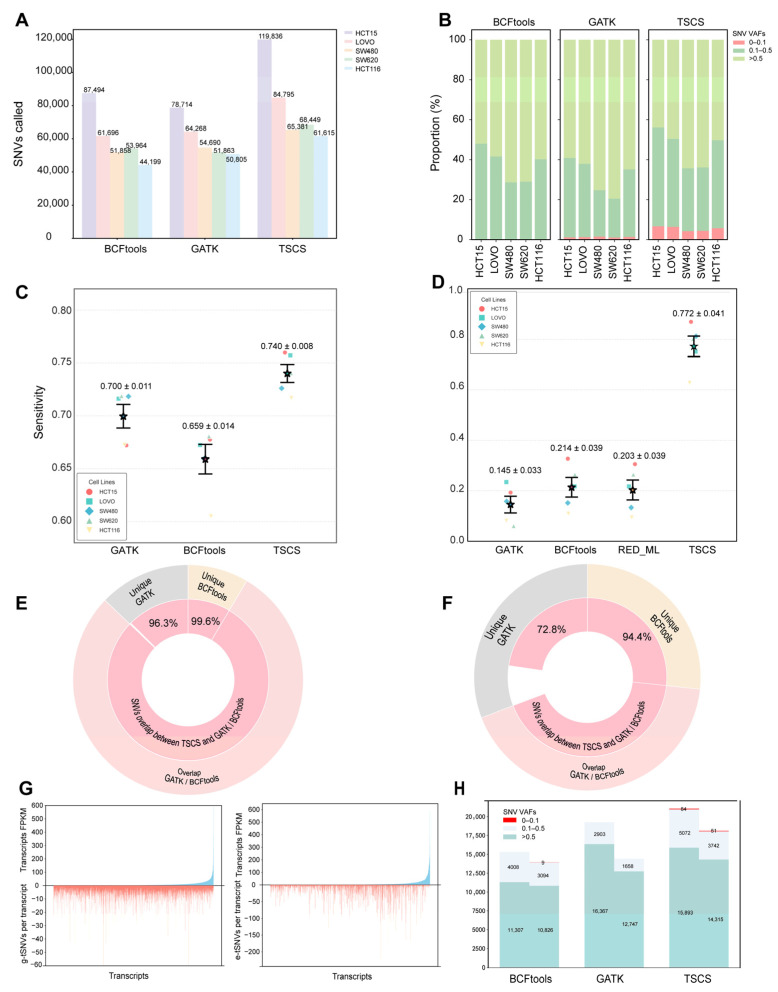
Comparison of transcript SNVs called from different software based on MGI RNA-seq data. (**A**) Comparison of SNVs called by GATK, BCFtools, and TSCS in five cell lines. (**B**) In view of SNVs VAFs, comparison of SNVs called by GATK, BCFtools, and TSCS in five cell lines. (**C**) Comparison of RS:g-tSNVs per cell line derived from GATK, BCFtools, and TSCS in five cell lines. Points show individual samples; stars and error bars indicate mean ± SEM (*n* = 5). (**D**) Comparison of RS:e-tSNVs per cell line derived from GATK, BCFtools, RED_ML, and TSCS in five cell lines. Points show individual samples; stars and error bars indicate mean ± SEM (*n* = 5). (**E**) Overlap status of total g-tSNVs elicited from GATK and BCFtools against that from TSCS in all the five cell lines. Outer ring represents g-tSNVs overlap between GATK and BCFtools and inner ring means g-tSNVs overlap between TSCS and GATK or BCFtools. (**F**) Overlap status of total e-tSNVs elicited from GATK and BCFtools against that from TSCS in all the five cell lines. (**G**) Distribution of abundance per transcript and g-tSNVs detected per transcript (**left**), as well as e-tSNVs detected per transcript (**right**) from TSCS in HCT15. (**H**) Comparation of the SNVs recognized by BCFtools, GATK, and TSCS, which are derived from the public database and are commonly found in over 70% samples.

Figure 2A summarizes the total SNVs identified by GATK, BCFtools, and TSCS across the five cell lines using MGI data. While all three tools yielded SNVs with similar distribution patterns, TSCS consistently detected a greater number of variants, increasing the total yield of transcriptomic SNVs by 31.83% compared with the average of the other methods, with g-tSNV and e-tSNV counts exceeding those of conventional methods by more than 1-fold and 2-fold, respectively. A comparative analysis of VAF distributions revealed that TSCS recovered a higher proportion of low-VAF SNVs (Figure 2B). Furthermore, Figure S4 highlights a pronounced divergence between e-tSNVs and g-tSNVs: e-tSNVs were predominantly low frequency (>70% at VAF < 0.5; only 4% at VAF = 1.0), whereas g-tSNVs showed higher VAF values (40% at VAF = 1.0; 26% below 0.5). We defined recognition sensitivity for g-tSNVs (RS:g-tSNVs) as the ratio of transcript-detected g-tSNVs to the expressed genomic SNVs, i.e., genomic SNVs located in coding regions that are covered by both genomic and transcriptomic sequencing data. As shown in Figure 2C, TSCS achieved moderately higher RS:g-tSNVs across all cell lines compared with GATK and BCFtools, with an average recall of 74% versus 70% for GATK and 65% for BCFtools, representing absolute improvements of 4 and 8 percentage points, respectively. For e-tSNVs, due to the absence of a gold-standard set, we defined a PacBio-validated reference set (read depth ≥ 100, variant reads ≥ 30). RS:e-tSNVs was then calculated as the recall rate, i.e., the proportion of e-tSNVs in this reference set that were successfully detected by each tool. TSCS demonstrated substantially higher RS:e-tSNVs than the other tools (Figure 2D), achieving an average e-tSNV recall of 77% compared with 14% for GATK, 21% for BCFtools, and 20% for RED_ML, corresponding to absolute improvements of 62, 51, and 51 percentage points, respectively, indicating particularly enhanced sensitivity for e-tSNV detection. Overall, TSCS demonstrates superior capability in identifying both g-tSNVs and e-tSNVs from the MGI data, at least in cell lines.

Overlap rates of total transcript SNVs, g-tSNVs, and e-tSNVs between GATK and BCFtools were first evaluated across five cell lines, showing 79% agreement for g-tSNVs but only 43% for e-tSNVs (Figure S5A,D), indicating poor consistency in e-tSNV calling. In contrast, TSCS overlapped with 99.4% and 99.9% of g-tSNVs called by GATK and BCFtools, respectively, and captured 88.56% and 97.8% of their e-tSNVs (Figure S5B,C,E,F). Notably, among the g-tSNVs and e-tSNVs uniquely identified using GATK or BCFtools, TSCS recovered 96.3% and 99.6% of g-tSNVs, and 72.8% and 94.4% of e-tSNVs, respectively (Figure 2E,F), demonstrating its superior comprehensiveness. Furthermore, we assessed whether SNV detection was influenced by transcript abundance. As shown in Figure 2G, both g-tSNVs and e-tSNVs called by TSCS were evenly distributed across transcripts regardless of expression level, indicating that variant calling with TSCS is not affected by transcript abundance.

To evaluate the robustness and generalizability of TSCS, we analyzed CRC tissue RNA-seq data from NCBI SRA (accessions SRP303779 and SRP303082) [45] using the same bioinformatic workflow. Supplementary Figure S6 summarizes transcript SNV detection rates across clinical samples, showing that TSCS identified markedly more high-detection-rate SNVs than GATK or BCFtools in both adjacent and tumor tissues. We further examined SNVs co-detected in over 70% of samples by all three tools across both tissue types. As shown in Figure 2H, TSCS yielded more high-quality transcript SNVs across three VAF ranges compared with the other methods, except at VAF > 0.5 where GATK performed comparably. These results strongly support that TSCS robustly captures more transcript SNVs, particularly at low VAFs, and demonstrate its potential for reliable SNV identification in tissue-derived data.

We further evaluated TSCS on PacBio RNA-seq data from the five cell lines. As summarized in Supplementary Figure S7, TSCS increased the total yield of transcriptomic SNVs by 71.89% compared with the average of the other tools. For gSNV detection, it improved the mean recall from 54.56% to 70.69%, representing an absolute gain of 15.77 percentage points over GATK, BCFtools, and Longshot. For tSNV detection, TSCS elevated the average recall from 28.82% to 54.54%, an absolute improvement of 25.72 percentage points compared with GATK, BCFtools, Longshot, and RED_ML. TSCS consistently outperformed existing tools in recognition sensitivity and overlap rates for both g-tSNVs and e-tSNVs, mirroring trends observed in the MGI data. Notably, the performance improvement achieved by TSCS was more pronounced on PacBio data than on MGI data, despite the lower number of transcript SNVs typically detected from long-read sequencing.

### 3.3. Verification of Transcript SNVs and Their Translated Products in Cell Lines

The transcript SNVs by TSCS were further verified through three complementary experimental approaches: Sanger sequencing to confirm the target SNV segments [46], ribosome-nascent chain complex sequencing (RNC-seq) to detect the SNVs on the translational track [47,48,49], and mass spectrometry to identify the translated variant peptides [50,51,52,53].

**Figure 3:**
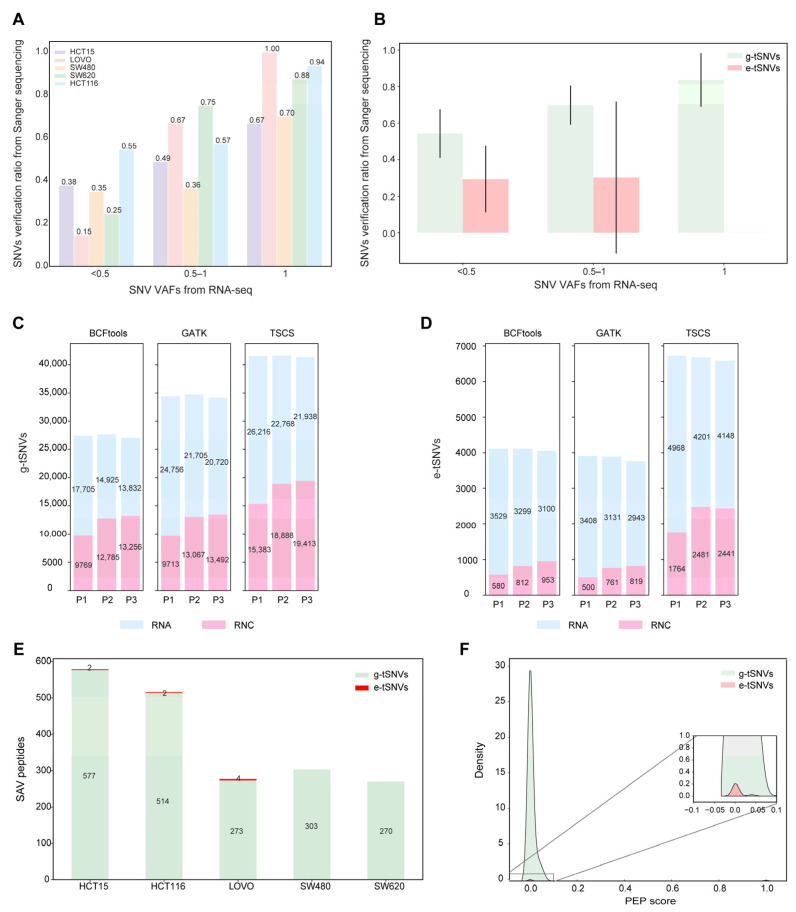
Verification of transcript SNVs and proteins SAVs using Sanger sequencing, RNC-seq, and mass spectrometry in five cell lines. (**A**) Distribution of the verification rates to transcript SNVs using Sanger sequencing through the SNVs detected using Sanger against that selected from the transcript SNVs library in the five cell lines. (**B**) Verification rates of g-tSNVs and e-tSNVs using Sanger sequencing across five cell lines. Error bars indicate the standard deviation across cell lines for each group. (**C**,**D**) Verification of g-tSNVs and e-tSNVs in HCT116 (*n* = 3) using RNC-seq. (**E**) The identified peptides corresponding to SAVs elicited from g-tSNVs and e-tSNVs in the five cell lines. (**F**) Distribution of PEP scores for all the peptides identified using LC MS/MS to view the peptide identification quality.

To independently validate transcript SNVs identified using TSCS, we performed Sanger sequencing on mRNA amplicons from 25 randomly selected gene segments across the five colorectal cancer cell lines. The validation confirmed 173, 33, 47, 42, and 50 SNVs in HCT15, LoVo, SW480, SW620, and HCT116, respectively. Among these, 121, 8, 31, 4, and 23 were verified as e-tSNVs. The overall validation rates for TSCS-predicted SNVs reached 40%, 33%, 37%, 45%, and 58% across the respective cell lines. As shown in Figure 3A, the validation rate by Sanger sequencing increased with higher VAF values (based on MGI data). Further analysis of g-tSNVs and e-tSNVs across three VAF categories (Figure 3B) revealed that g-tSNVs showed validation rates proportional to VAF, whereas e-tSNVs were primarily detected in the lower VAF category, with few in higher VAF ranges, consistent with the distribution shown in Figure S4. The overall validation rate was approximately 30% for SNVs with VAF < 0.5, but exceeded 80% for those with a higher VAF. These results confirm the accuracy of TSCS-called SNVs and support the presence of both g-tSNVs and e-tSNVs in these cell lines, although lower validation rates for low-VAF variants indicate a need for larger clone numbers to fully verify these events [54].

The translatome, representing actively translating mRNAs bound to ribosomes, was profiled using RNC-seq to assess the translational potential of SNVs identified using TSCS, GATK, and BCFtools in HCT116 cells. As shown in Figure 3C,D, TSCS-derived g-tSNVs and e-tSNVs showed identification rates of 43% and 33%, respectively. In comparison, the rates for GATK were 35% and 18%, and 44% and 19% for BCFtools. These results not only confirm the presence of software-predicted SNVs but also unveil a significant portion of transcript SNVs on the translational track. Notably, while verification rates for g-tSNVs were comparable across tools, TSCS exhibited markedly higher validation rates for e-tSNVs, consistent with previous observations.

To further validate the translatability of TSCS-called SNVs, we performed peptide mass spectrometry. A total of 10,615, 6837, 5195, 4904, and 6505 nonsynonymous SNVs were annotated by TSCS in HCT15, LoVo, SW480, SW620, and HCT116, respectively. As shown in Figure 3E, the number of identified peptides correlated with the total SNV count per line (e.g., highest in HCT15), with g-tSNVs yielding higher identification rates than e-tSNVs, consistent with Sanger data (Figure 3B) and reflecting the challenge of detecting low-VAF e-tSNVs using MS [55,56]. Peptide identification rates were comparable across tools (TSCS: 5.5%, GATK: 5.6%, BCFtools: 6.5%; Figure S8). This suggests that the strategy of a peptide search upon MS/MS is workable through the peptide libraries containing SNVs generated from different SNVs calling programs. Posterior error probability (PEP) [57] score distributions (Figure 3F) confirmed high-quality matches for both g-tSNVs and e-tSNVs, with peaks near zero indicating confident identifications despite lower e-tSNV abundance.

Hence, the transcript SNVs recognized using TSCS across different cancer cell lines were rigorously verified through three experimental approaches, reinforcing the presence of g-tSNVs and e-tSNVs and leading to a clue that significant SNVs are translational and potentially functional.

### 3.4. Characterization of e-tSNVs in Cancer Cell Lines

**Figure 4:**
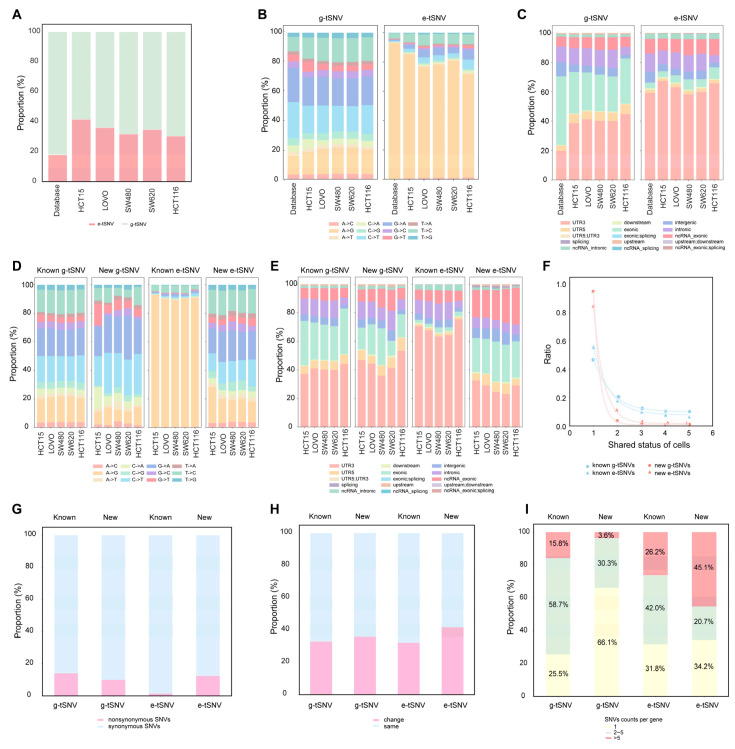
Characterization of the g-tSNVs and e-tSNVs called by TSCS in the five cell lines. (**A**) Comparison of the ratios of e-tSNVs versus g-tSNVs in public SNVs database and in the transcript SNVs called by TSCS in the five cell lines. (**B**) Comparison of the nucleotide substation-dependent SNV types in g-tSNVs and e-tSNVs in the public SNVs database and in the transcript SNVs called by TSCS in the five cell lines. (**C**) Comparison of the genomic regions of g-tSNVs and e-tSNVs in the public SNVs database and in the transcript SNVs called by TSCS in the five cell lines. (**D**) Comparison of the nucleotide substation-dependent SNV types of g-tSNVs and e-tSNVs between the known and new SNVs called by TSCS in the five cell lines. (**E**) Comparison of SNVs’ genomic regions between the known and new g-tSNVs and e-tSNVs. (**F**) The shared status of the known and new for g-tSNVs and e-tSNVs in the five cell lines. (**G**) Nonsynonymous SNVs of the total of known g-tSNVs and e-tSNVs, as well as of new g-tSNVs and e-tSNVs in all the cell lines. (**H**) Assessment of the changes in amino acid properties induced by nonsynonymous g-tSNVs and e-tSNVs, known or new, in all the cell lines. (**I**) Distribution of nonsynonymous SNVs per gene for g-tSNVs and e-tSNVs, known or new.

We compiled a non-redundant set of public SNVs by merging g-tSNVs from dbSNP [58] and COSMIC [59] with e-tSNVs from REDIportal and DARNED for comparative analysis. As shown in Figure 4A, e-tSNVs constituted 18% of the total SNVs in this public dataset (921,841/5,147,327), whereas TSCS identified a notably higher proportion (30–42%) across the five cell lines (Table S3), indicating that e-tSNVs represent a larger fraction of transcriptomic variation than previously recognized, with stable proportions observed in cancer cells. Analysis of SNV types (Figure 4B) revealed that both public and TSCS g-tSNVs were dominated by C>T, G>A, A>G, and T>C changes. However, while public e-tSNVs were overwhelmingly A-to-I(G) (93%), TSCS-called e-tSNVs included substantial non-A-to-I changes (>20%), consistent with reports of more diverse RNA editing [7,60,61]. Regarding genomic distribution, both public and TSCS-derived SNVs exhibited similar patterns: g-tSNVs showed less than 40% localization in 3′UTR, while e-tSNVs were enriched in 3′UTR (>60%; Figure 4C). This trend persisted among individual base substitutions: g-tSNVs maintained ∼40% UTR3 localization across all types, whereas among e-tSNVs, A-to-I(G) and T-to-C events were highly enriched in UTR3 (>60%), while other substitutions showed less UTR3 preference (<40%; Figure S9).

On average, 92% of g-tSNVs were overlapped with the public g-tSNVs databases from dbSNP and COSMIC, and 80% of e-tSNVs were overlapped with the e-tSNVs databases in REDIportal and DARNED. These overlapped SNVs were termed as known variants. The remaining comprised 21,284 new g-tSNVs and 18,173 new e-tSNVs, which were distributed cell-specifically across HCT15, LoVo, SW480, SW620, and HCT116. Only 316 new g-tSNVs and 503 new e-tSNVs were shared across ≥80% of the cell lines. As shown in Figure 4D, new g-tSNVs closely resembled known g-tSNVs in SNV type profiles, whereas new e-tSNVs exhibited markedly different patterns. While known e-tSNVs were dominated by A-to-I(G) (>90%), new e-tSNVs showed considerable diversity, with A-to-I(G) accounting for less than 20% and numerous other base changes well represented. This result aligns well with the deduction from Figure 4B that TSCS improvement in e-tSNVs discovery appears to dig SNV types with more base alterations. A genomic region analysis (Figure 4E) indicated that new g-tSNVs retained distribution patterns similar to known g-tSNVs, but new e-tSNVs diverged significantly from known e-tSNV; known e-tSNVs were highly enriched in 3′UTR (>60%), whereas new e-tSNVs showed less than 40% in 3′UTR and increased presence in exonic regions. These findings indicate that new g-tSNVs resemble publicly documented variants in both type and genomic distribution, while novel e-tSNVs discovered by TSCS exhibit distinct characteristics from the known e-tSNVs; the new e-tSNVs are distributed across relatively larger genomics regions with many different base shifts.

To assess the generalizability of the observed e-tSNV diversity, we extended our analysis to multiple orthogonal datasets representing distinct experimental conditions (Figure S10). This included: (1) RNC-seq-validated SNVs from HCT116; (2) SNV calls from technical replicates of HCT15; and (3) SNVs identified from PacBio long-read data across all five cell lines. Critically, the patterns of SNV types and genomic distributions remained consistent across all these datasets and recapitulated the global profiles obtained from the MGI data analyzed by TSCS. These reproducible findings across translational profiling, technical replicates, and alternative sequencing technologies confirm that the observed diversity is non-random and firmly establishes that the expanded diversity of e-tSNVs represents a general biological characteristic rather than a technical artifact.

To assess the specificity of transcript SNVs, overlap ratios of known and new variants were evaluated across the five cell lines (Figure 4F). Unique known g-tSNVs and e-tSNVs (found in only one cell line) accounted for 47% and 55%, respectively, while unique new g-tSNVs and e-tSNVs were substantially higher at 94% and 84%, indicating strong cell-line specificity. The distribution patterns of SNV types and genomic regions for these unique and shared variants were consistent with those in Figure 4D,E (Figure S11C,D). There was evidence of new g-tSNVs and e-tSNVs that were specifically cell-dependent, therefore demonstrating that SNVs calling from TSCS indeed helps in the discovery of uncommon SNVs signals.

For further understanding of whether transcript SNVs lead to changes in amino acids, a nonsynonymous analysis of the total SNVs among the five cell lines was conducted and the results are detailed in Figure 4G. Generally, the known and new g-tSNVs had comparable rates (14% vs. 10%), whereas the known e-tSNVs showed a much lower nonsynonymous rate than the new e-tSNVs (1% vs. 13%), aligning with the typological diversity and broader genomics within the exons of new e-tSNVs. Based on a seven-category classification of amino acid properties [62], approximately 40% of nonsynonymous SNVs—across both known and new g-tSNVs and e-tSNVs—resulted in property-altering changes (Figure 4H). This proportion remained consistent despite substantial differences in variant numbers between known and new g-tSNVs (15,145 vs. 2126) and e-tSNVs (755 vs. 2284), indicating that the nonsynonymous SNVs’ differences seem irrelevant to the corresponding types of amino acids.

Moreover, looking at the distribution of nonsynonymous SNVs in each gene, the distribution of nonsynonymous SNVs per gene differed between g-tSNVs and e-tSNVs (Figure 4I). New e-tSNVs showed high clustering (45% with >5 SNVs/gene), prompting investigation into potential protein physicochemical effects like pI shift [63,64]. However, theoretical estimates revealed comparable pI changes across all variant types (Figure S11E), indicating clustered occurrences of new e-tSNVs without obvious property alterations in the corresponding proteins.

We assessed whether genes harboring new e-tSNVs were functionally enriched. Among the 8967 genes carrying nonsynonymous SNVs, only 294 contained exclusively new e-tSNVs. GO analysis of these 294 genes showed no significant enrichment.

## 4. Discussion

A large volume of evidence has demonstrated that SNVs are not only in genomes but also are generated during the transcriptional process, so-called RNA editing. How to accurately identify transcription-dependent SNVs, i.e., e-tSNVs, and clearly distinguish g-tSNVs and e-tSNVs have been debated for a long period due to inconsistent parameters across existing tools. Given the incomplete understanding of RNA editing mechanisms, a major challenge has been in developing accurate transcript SNV callers that avoid dependence on RNA-editing-specific parameters. To overcome this, we proposed that high-quality RNA-seq data alone could enable reliable SNV detection without relying on RNA editing features. Using integrated SNV calls from parallel MGI and PacBio sequencing as a high-confidence set, we developed TSCS, a random forest-based classifier. As shown in Figure 2C,D, TSCS achieved significantly higher recognition sensitivity for transcript SNVs compared with other tools across five cell lines, with particularly pronounced improvement for e-tSNVs over g-tSNVs. The high quality of TSCS-called variants is supported by multiple lines of evidence: most SNVs identified by GATK and BCFtools were also detected by TSCS (Figure 2E,F); TSCS-unique SNVs showed similar abundance and detection frequency profiles to the consensus variants; and critically, orthogonal validation through Sanger sequencing, RNC-seq, and mass spectrometry confirmed the detectability and authenticity of TSCS-derived SNV signals. Another concern is the biological plausibility of Non-Canonical RNA editing because several studies have claimed the diversity of genuine RNA editing events, whereas some cautioned that the editing sites might have arisen from pervasive technical artifacts [21,22]. To systematically assess whether the diverse RNA editing variants identified in our study were related to technical artifacts or not, we performed a comprehensive series of artifact-exclusion analyses across four variant sets (new e-tSNVs, known e-tSNVs, new g-tSNVs, and known g-tSNVs), including assessments of read-end and strand biases, splice-junction enrichment, mis-mapping to homologous sequences/paralogs, and chemical damage artifacts (e.g., oxidation-induced G>T transversions) during library preparation. As shown in Figure S12, the variant sets, including the new e-tSNVs, did not exhibit significant enrichment for the most common technical artifacts, thereby supporting the biological relevance of the detected RNA editing diversity. An exception was observed for chemical damage artifacts during library preparation, where the average ArtQ score was 14.3, below the ideal threshold of >30, indicating an elevated background level of G>T errors. However, G>T signals did not dominate the variant sets, comprising less than 10% of all called variants, and no bias from other technical artifacts was observed among these G>T variants. Therefore, the relatively low ArtQ score appears to have only a limited impact on variant identification. Collectively, this evidence supports that TSCS-called transcript SNVs are reasonably acceptable in furthering the relevant characteristic study and a good replenishment to the previous databases in this frontier.

We have realized that the Sanger verification to e-tSNVs at a low rate may raise concerns about potential false positives among the TSCS-predicted variants. According to our view, the concerns are partially relieved. First of all, Sanger sequencing is inherently challenged by stochastic dropout during PCR/cloning of low-frequency transcripts, leading to the underdetection of genuine variants. Our data showed that verification rates were dependent on VAF in both g-tSNVs and e-tSNVs (Figure 3B). If the verification of g-tSNVs with low VAF is acceptable, there is no reason to reject the verified e-tSNVs with a low frequency. Secondly, 95% of SNVs with low VAF discovered by TSCS and randomly picked up for Sanger sequencing are overlapped with public datasets, indicating that the presence of such SNVs is not solely perceived by TSCS. And thirdly, multiple approaches, such as Sanger sequencing, RNC-seq, and mass spectrometry, were used for the verification of the predicted SNVs from TSCS, and achieved the supportive evidence for each other. For instance, chr3_ 44749267 e-tSNV was not verified by Sanger sequencing, but was detected by RNC-seq.

RNA editing-mediated transcript-dependent SNVs are well established, with adenosine-to-inosine (A-to-I) conversion catalyzed by ADARs representing a canonical type of e-tSNVs [17] that occurs in both coding and non-coding regions, some leading to nonsynonymous amino acid changes [19] and being implicated in various cancers, such as glioblastoma, liver cancer, esophageal carcinoma, breast, colorectal, gastric cancer, and so on. For instance, A-to-I editing in AZIN1 promotes oncogenic signaling and nuclear translocation in liver cancer [65,66], while A-to-I editing in CDC14B modulates the Skp2/p21/p27 pathway in glioblastoma [67]. Beyond A-to-I, C-to-U editing mediated by APOBEC enzymes can disrupt protein coding by introducing premature stop codons or altering start sites, contributing to tumorigenesis [18,68,69]. Although diverse RNA editing types have been reported—such as five in human fibroblasts [70], nine in brain tissues [71], and up to twelve in B cells and cancer cell lines [60,61]—a comprehensive atlas is still lacking. In this study, TSCS-based analysis of five CRC cell lines revealed that e-tSNVs consistently accounted for approximately 40% of all transcript SNVs, suggesting a previously underreported diversity in both type and genomic distribution. Although A-to-I editing remained dominant (~80% of e-tSNVs), a substantial fraction (~20%) comprised other subtypes—including C-to-U, G-to-A, and T-to-C—distributed across 3′UTR, exonic, and ncRNA exonic regions (Figure 4B,D,E). The reliability of these findings is supported by three lines of evidence: detection criteria were consistent with those for known variants; patterns were reproducible across all five cell lines and in multiple orthogonal datasets representing distinct experimental conditions, suggesting that the observation is not random events; and a subset was validated via orthogonal assays. It is important to note a technical limitation inherent in non-strand-specific RNA-seq data. Even though computational strand assignment could correct most alignments, it cannot fully resolve overlapping antisense transcription. Consequently, a portion of the reported “non-canonical” edits, particularly T>C and G>A variants, could represent canonical A-to-I editing events occurring in antisense transcripts. Therefore, the precise characterization towards the novel and non-canonical RNA editing events with biological significance would be required for future validation with strand-specific sequencing. Additionally, theoretical explanations for these novel RNA editing events remain to be fully elucidated. As mentioned above, the deamination reaction well explains the conversion of A-to-I and C-to-U; however, the reaction mechanisms responsible for G-to-A and T-to-C conversions remain to be addressed in future studies. In summary, TSCS represents a significant advance in transcriptomic SNV screening, providing novel insights into RNA editing biology and its superior performance validated in clinical RNA-seq samples, and demonstrating its robust capability to uncover clinically relevant e-tSNVs that may illuminate previously unrecognized mechanisms in cancer pathogenesis, offering new avenues for understanding functional SNV involvement in tumor development.

## 5. Conclusions

This study developed TSCS, an integrated machine learning classifier that combines short-read and long-read RNA sequencing data to accurately distinguish transcript SNVs. Applied to colorectal cancer cell lines, TSCS demonstrated a superior performance over existing tools, significantly increasing the detection of both genomic variants and RNA editing events. Our results revealed that RNA editing constitutes approximately 40% of all transcript SNVs in these cells, with 80% of these e-tSNVs representing known RNA editing types and the remaining 20% comprising novel, previously unclassified variants that exhibit distinct mutational and genomic distribution patterns. Despite persistent challenges in validating low-VAF variants, orthogonal experimental approaches support the reliability of TSCS. In summary, this work establishes a robust analytical framework for the systematic discovery and characterization of cancer-related RNA editing, providing a foundation for future functional studies.

## Data Availability

The RNA-seq data underlying this article are available in the CNGB Sequence Archive (CNSA) of the China National GeneBank DataBase (CNGBdb) with accession number CNP0006938. The mass spectrometry proteomics data have been deposited to the ProteomeXchange Consortium (https://proteomecentral.proteomexchange.org, accessed on 25 January 2026) via the iProX partner repository with the dataset identifier PXD061293. All the codes are available on the GitHub repository at https://github.com/zhangxia-prog/TSCS (accessed on 25 January 2026).

## Supplementary Materials

The following supporting information can be downloaded at: https://www.mdpi.com/article/10.3390/biom16020211/s1, Figure S1. Systematic comparison of MGI and PacBio platforms for transcriptomic SNV detection in five colorectal cancer cell lines. Figure S2. Feature importance ranking of transcriptomic SNVs characteristics used for model training. Figure S3. Comparative ROC analysis of five machine learning algorithms for transcriptomic SNV classification. Figure S4. (A) Comparative analysis of VAF distributions between e-tSNVs and g-tSNVs identified by TSCS across five cell lines. Error bars indicate the standard deviation (SD) across cell lines for each VAF-type group. (B) Fine-scale VAF distribution of g-tSNVs (VAF < 0.5) in the HCT15 cell line. The chart shows the percentage of g-tSNVs falling into each of the specified VAF intervals. (C) Fine-scale VAF distribution of e-tSNVs (VAF < 0.5) in the HCT15 cell line. The chart shows the percentage of e-tSNVs falling into each of the specified VAF intervals. Figure S5. Comparative analysis of SNVs detection concordance among GATK, BCFtools, and TSCS across five cell lines. Figure S6. Transcript SNV detection rates in CRC patient-matched tissues. Figure S7. Comparison of transcript SNVs called from different software based on PacBio RNA-seq data. Figure S8. Peptide identification rates for nonsynonymous transcript SNVs detected by GATK, BCFtools, and TSCS. Figure S9. Genomic regions distribution of all the individual base substitutions in g-tSNVs and e-tSNVs called by TSCS across five cell lines. Figure S10. Characterization of the g-tSNVs and e-tSNVs called by TSCS across orthogonal datasets under varied experimental conditions. Figure S11. Characterization of the shared g-tSNVs and e-tSNVs called by TSCS in the five cell lines. Figure S12. Systematic artifact-exclusion analysis of four variant sets (new e-tSNVs, known e-tSNVs, new g-tSNVs, and known g-tSNVs). File S1: Parameter settings of GATK and BCFtools; Table S1. Primary SNVs; Table S2. TSCS-identified SNVs; Table S3. g-tSNVs and e-tSNVs; Table S4. Sanger primer.
