# Supplementary material for: A Global Assessment of the Transcription-Dependent Single Nucleotide Variants Relies on the Characteristics of RNA-Sequencing Technologies
Source: Biomolecules. 2026 Jan 29;16(2):211. doi: 10.3390/biom16020211 (PMC12937670; doi:10.3390/biom16020211)
Supplement: Supplementary file 1 [file biomolecules-16-00211-s001.zip › Supplementary Figures.pdf]

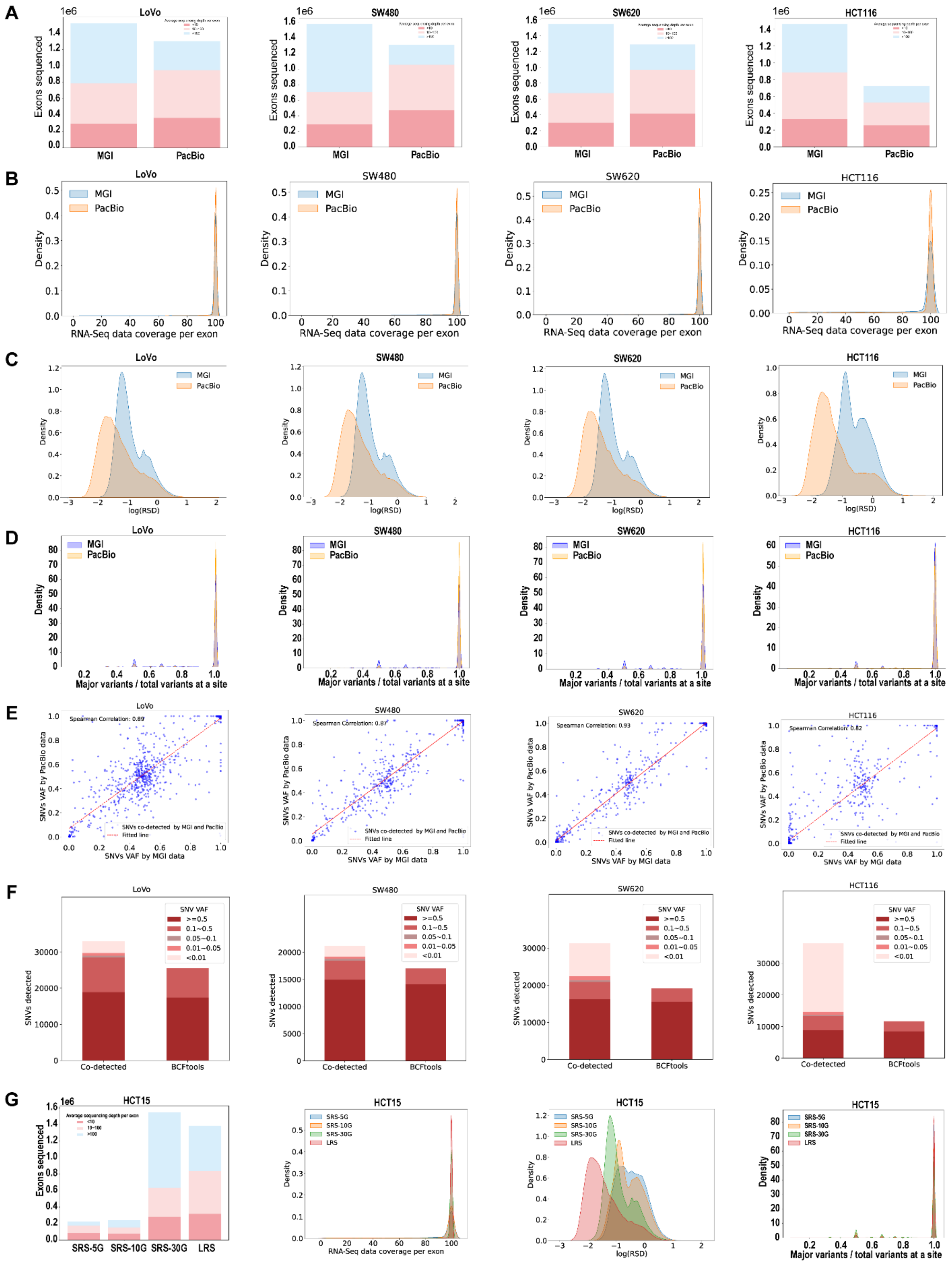

Supplementary Figure S1. Systematic comparison of MGI and PacBio platforms for transcriptomic SNV detection in five colorectal cancer cell lines. (A) Comparison of the identified exons between two sequencing datasets according to the sequencing depth per exon in LoVo,

SW480, SW620, and HCT116 cell lines. (B) Comparison of the sequencing coverage per exon between two sequencing datasets in LoVo, SW480, SW620, and HCT116 cell lines. (C) RSD distribution of the sequencing depth per exon in the two sequencing datasets in LoVo, SW480, SW620, and HCT116 cell lines. (D) Evaluation of data consistency for SNVs identified by two different sequencing approaches in LoVo, SW480, SW620, and HCT116 cell lines. (E) Correlation of VAF for the SNVs co-identified by two different sequencing approaches using Spearman correlation analysis in LoVo, SW480, SW620, and HCT116 cell lines. (F) Comparison of the SNVs extracted by the two different calling approaches according to the SNVs at different levels of VAF in LoVo, SW480, SW620, and HCT116 cell lines. And (G) Comparison of SNV profiles derived from Cross-batch of SRS and LRS RNA-seq of HCT15.

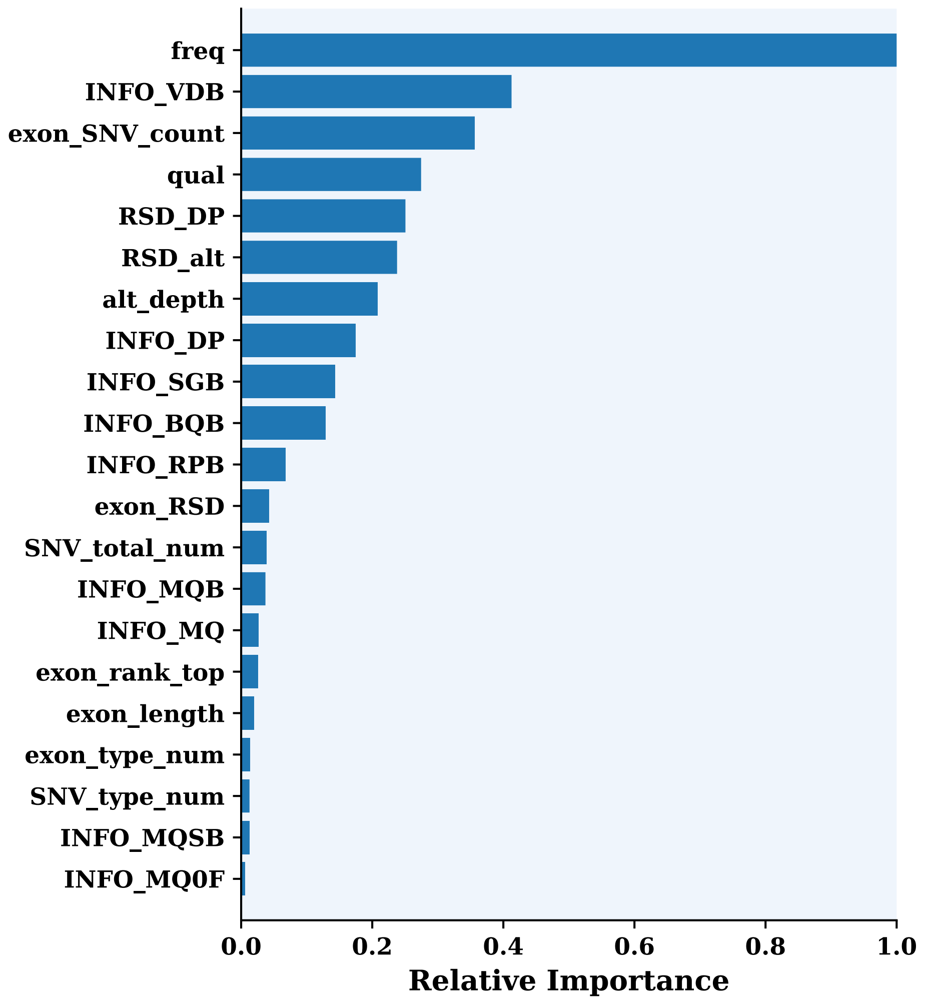

Supplementary Figure S2. Feature importance ranking of transcriptomic SNVs characteristics used for model training.

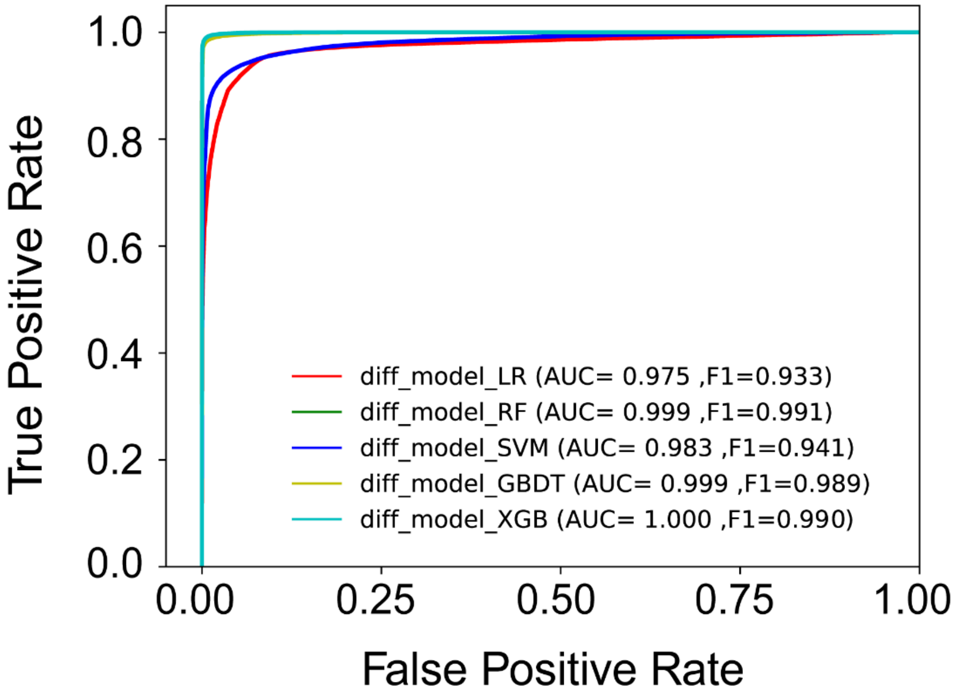

Supplementary Figure S3. Comparative ROC analysis of five machine learning algorithms for transcriptomic SNV classification. Performance metrics (F1 scores) demonstrate discrimination efficacy.

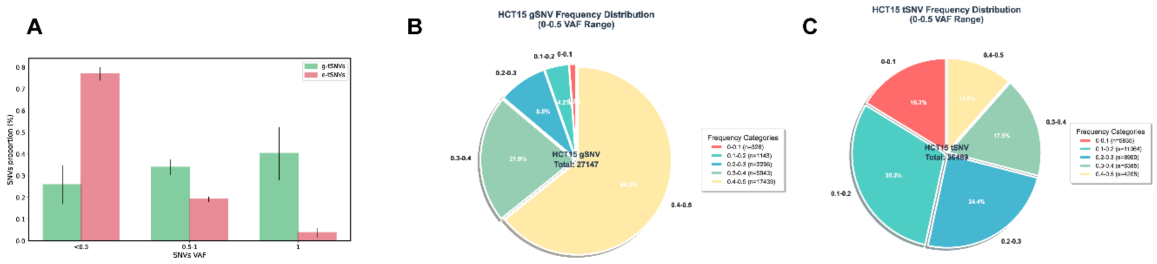

Supplementary Figure S4. (**A)** Comparative analysis of VAF distributions between e-tSNVs and g-tSNVs identified by TSCS across five cell lines. Error bars indicate the standard deviation (SD) across cell lines for each VAF-type group. (**B)** Fine-scale VAF distribution of g-tSNVs (VAF < 0.5) in the HCT15 cell line. The chart shows the percentage of g-tSNVs falling into each of the specified VAF intervals. (**C)** Fine-scale VAF distribution of e-tSNVs (VAF < 0.5) in the HCT15 cell line. The chart shows the percentage of e-tSNVs falling into each of the specified VAF intervals.

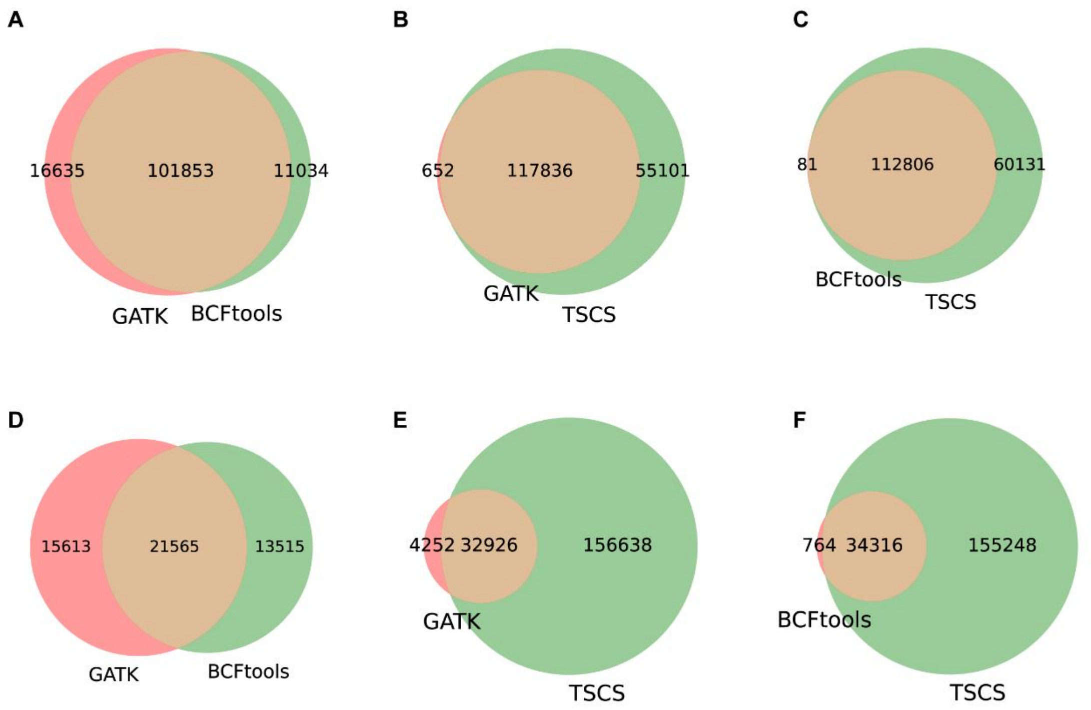

Supplementary Figure S5. Comparative analysis of SNVs detection concordance among GATK, BCFtools and TSCS across five cell lines. Venn diagrams depict (A-C) g-tSNV and (D-F) e-tSNV overlaps between: (A,D) GATK-BCFtools, (B,E) GATK-TSCS, and (C,F) BCFtools-TSCS pairwise comparisons.

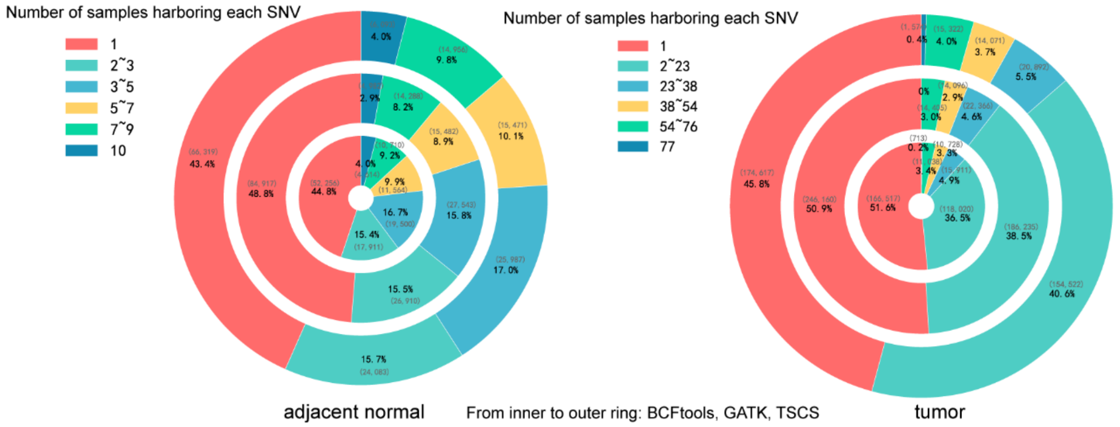

Supplementary Figure S6. Transcript SNV detection rates in CRC patient-matched tissues. Three-layer radial plots compare variant calling performance of BCFtools (inner), GATK (middle), and TSCS (outer) between normal (left) colorectal mucosa and paired tumor (right) specimens.

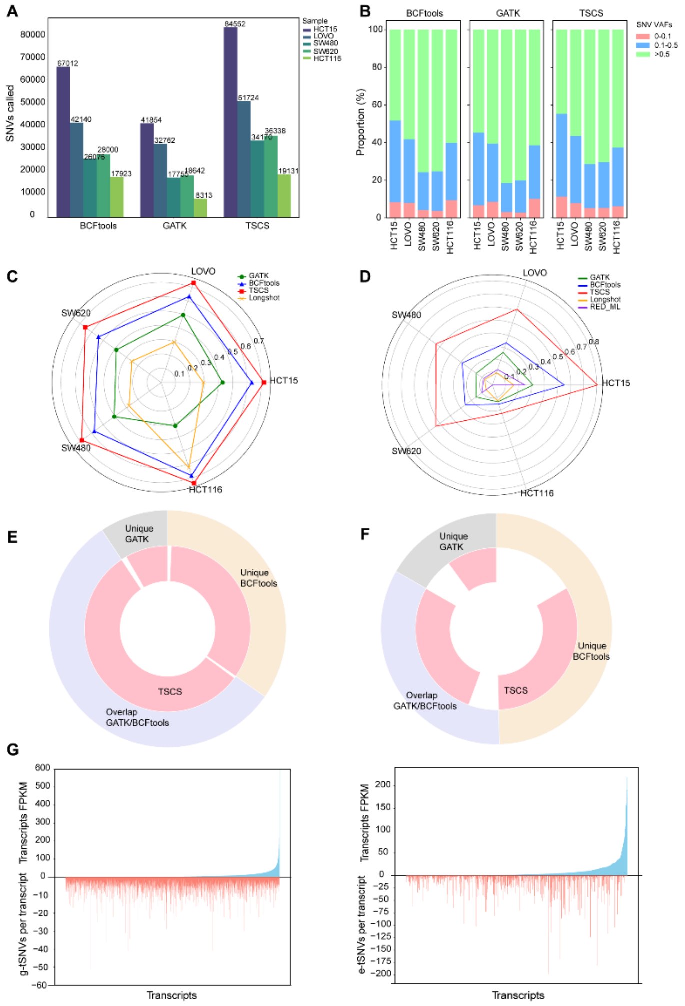

Supplementary Figure S7. Comparison of transcript SNVs called from different software based on PacBio RNA-seq data. (**A)** Comparison of SNVs called by GATK, BCFtools, and TSCS in five cell lines. (**B)** In view of SNVs VAF, comparison of SNVs called by GATK, BCFtools, and TSCS in five cell lines. (**C)** Radar plot represents comparison of ratios of total transcript g-tSNVs verse total genome SNVs per cell line derived from GATK, BCFtools, and TSCS in five cell lines. (**D)** Radar plot represents comparison of ratios of the transcript e-tSNVs called by a software matched with experimental e-tSNVs verse total experimental e-tSNVs in five cell lines. (**E)** Overlap status of total g-tSNVs elicited from GATK and BCFtools against that from TSCS in all the five cell lines. (**F)** Overlap status of total e-tSNVs elicited from GATK and BCFtools against that from TSCS in all the five cell lines. And (**G)** Distribution of abundance per transcript and g-tSNVs detected per transcript (left) as well as e-tSNVs detected per transcript (right) from TSCS in HCT15.

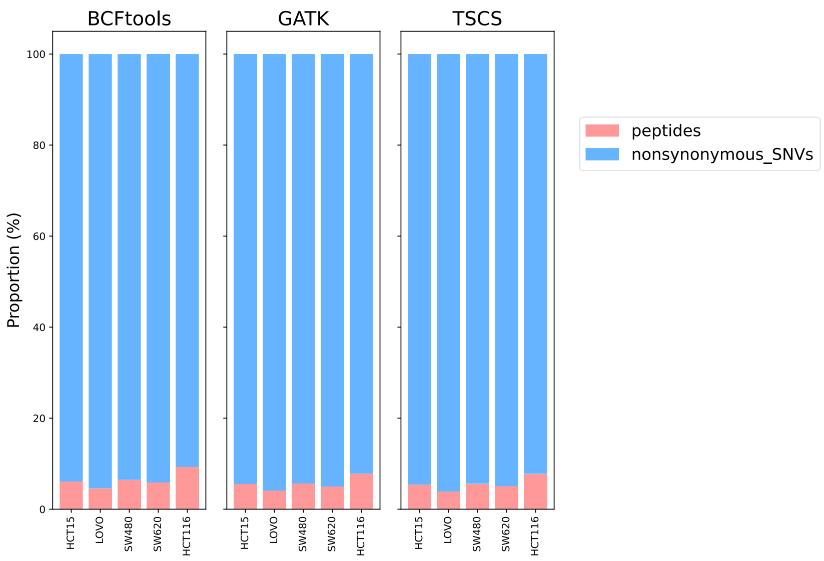

Supplementary Figure S8. Peptide identification rates for nonsynonymous transcript SNVs detected by GATK, BCFtools, and TSCS.

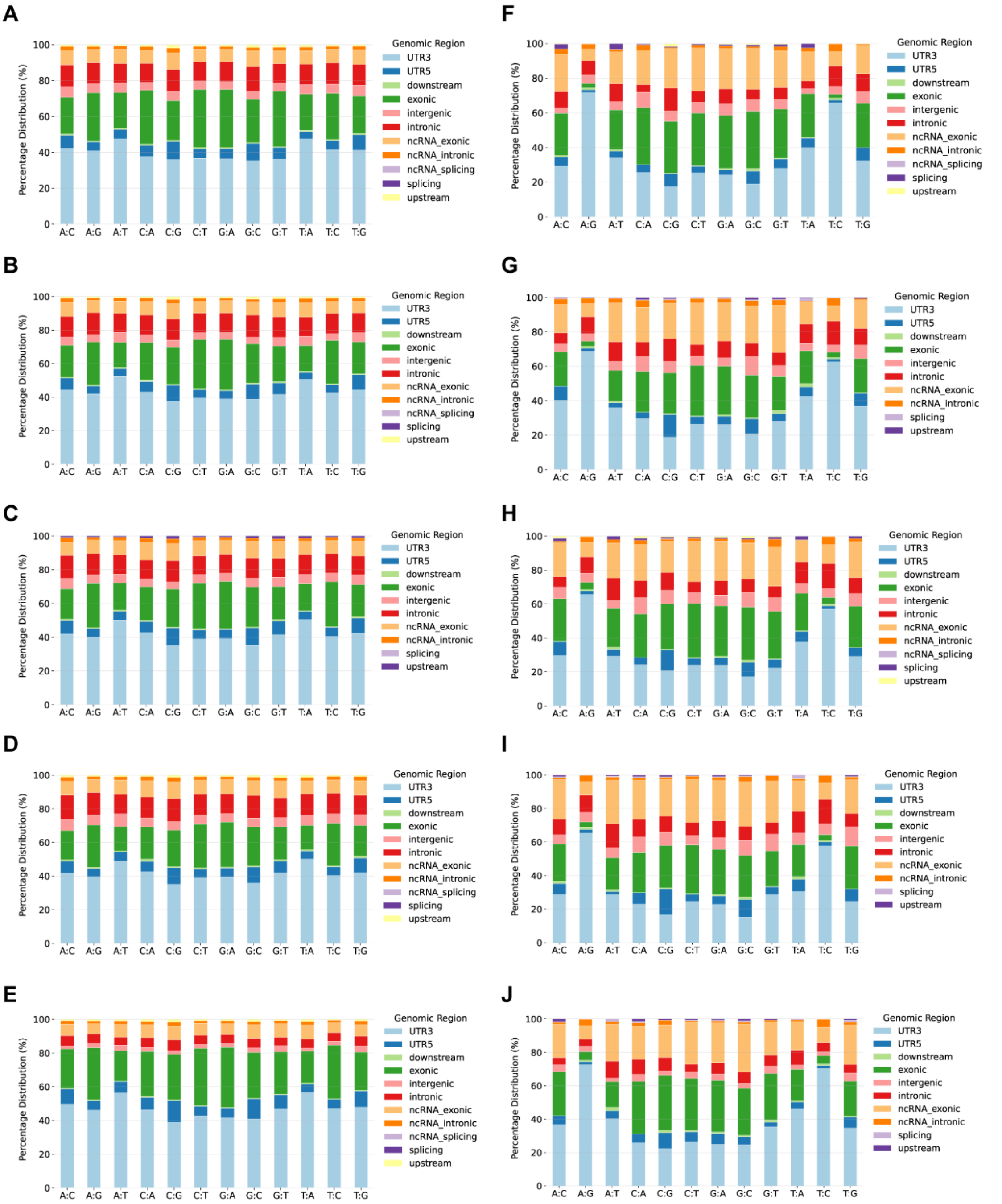

Supplementary Figure S9. Genomic regions distribution of all the individual base substitutions in g-tSNVs and e-tSNVs called by TSCS across five cell lines. Distributions of g-tSNVs **(A-E)** and e-tSNVs **(F-J)** across **(A,F)** HCT15, **(B,G)** LoVo, **(C,H)** SW480, **(D,I)** SW620, and **(E,J)** HCT116.

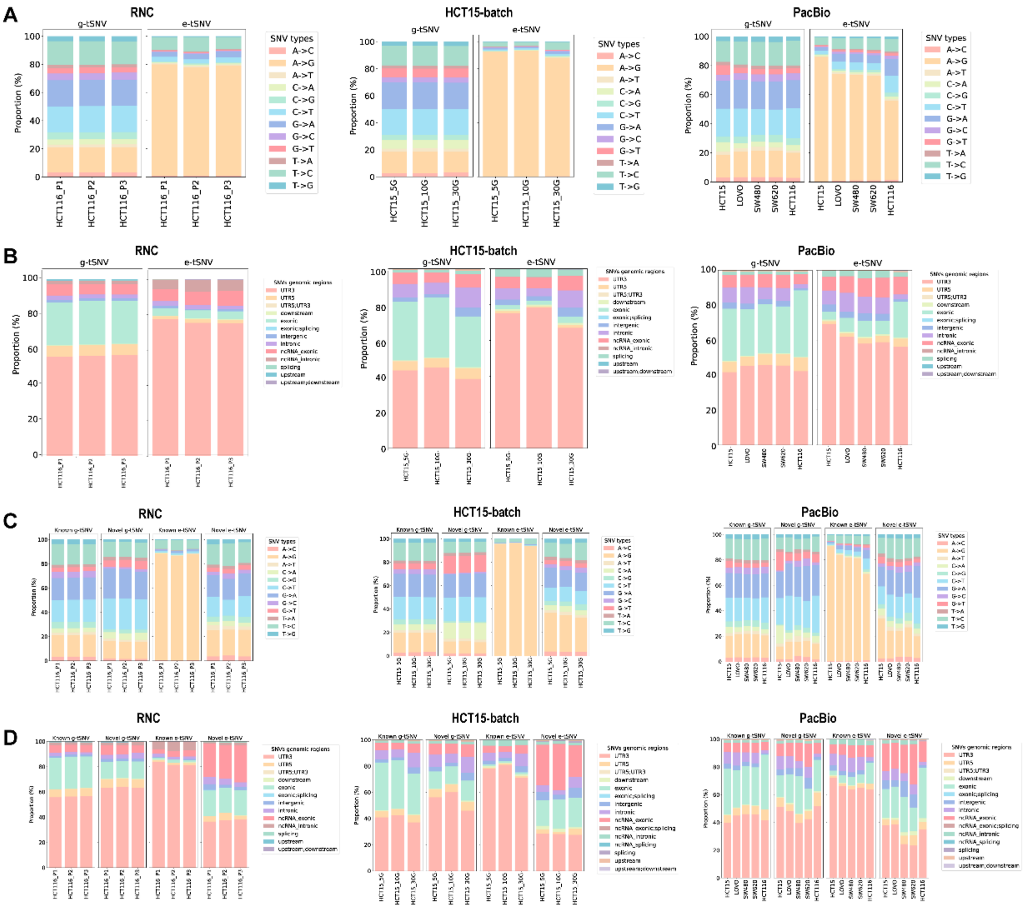

Supplementary Figure S10. Characterization of the g-tSNVs and e-tSNVs called by TSCS across orthogonal datasets under varied experimental conditions. (A) Comparison of the nucleotide-substation dependent SNV types in g-tSNVs and e-tSNVs derived from three independent sources: (i) RNC-seq-validated SNVs in HCT116, (ii) technical replicate SRS RNA-seq SNV calls in HCT15, and (iii) LRS PacBio RNA-seq SNV calls across five cell lines. (B) Comparison of the genomic regions of g-tSNVs and e-tSNVs derived from the same datasets as in (A). (C) Comparison of the nucleotide-substation dependent SNV types of g-tSNVs and e-tSNVs between the known and new SNVs derived from the same datasets as in (A). And (D) Comparison of SNVs genomic regions between the known and new g-tSNVs and e-tSNVs derived from the same datasets as in (A).

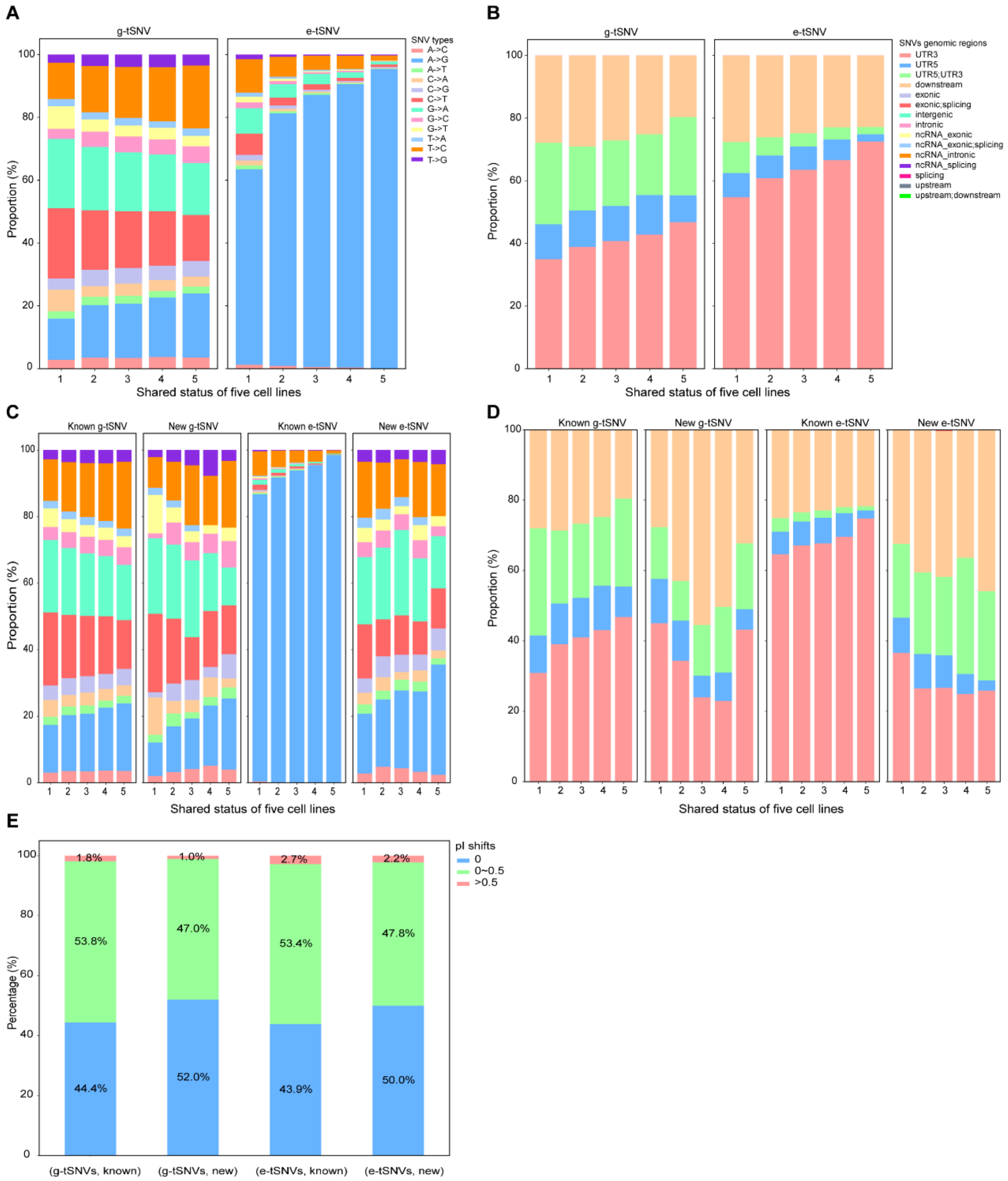

Supplementary Figure S11. Characterization of the shared g-tSNVs and e-tSNVs called by TSCS in the five cell lines. (**A)** Comparison of the base-substation dependent SNV types in shared g-tSNVs and e-tSNVs called by TSCS in the five cell lines. (**B)** Comparison of the genomic regions of shared g-tSNVs and e-tSNVs called by TSCS in the five cell lines. (**C)** Comparison of the base-substation dependent SNV types of shared g-tSNVs and e-tSNVs between the known and new SNVs called by TSCS in the five cell lines. (**D)** Comparison of SNVs genomic regions between the known and new shared g-tSNVs and e-tSNVs. And (**E)** Assessment of the shifts in proteins pI induced by SAVs corresponding to the nonsynonymous g-tSNVs and e-tSNVs, known or new, in all the cell lines.

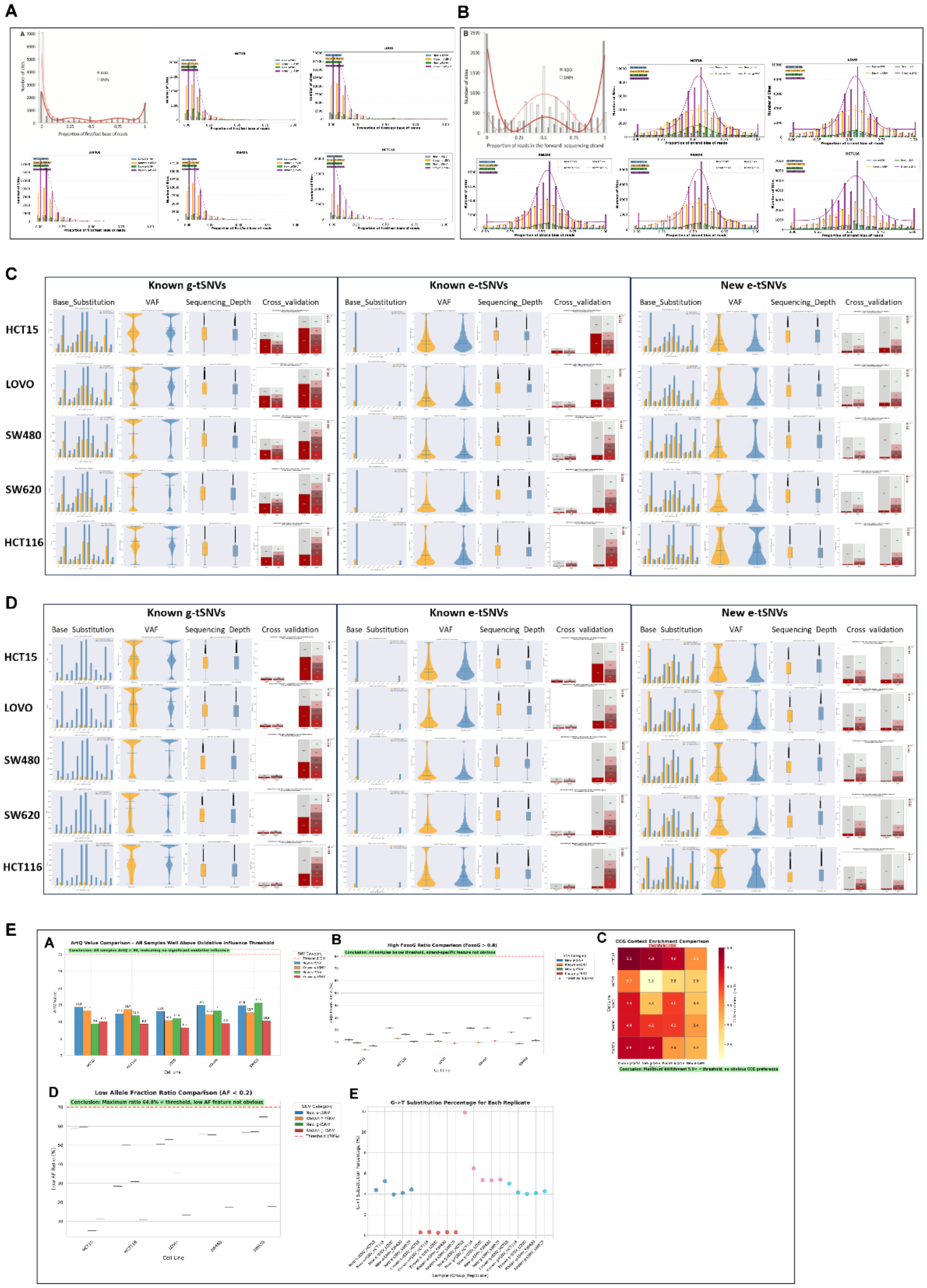

Supplementary Figure S12. Systematic artifact-exclusion analysis of four variant sets (new e-tSNVs, known e-tSNVs, new g-tSNVs, and known g-tSNVs). These analyses included evaluations of: **(A)** read-end and **(B)** strand biases; **(C)** splice-junction enrichment; **(D)** potential mis-mapping to homologous sequences or paralogs; and **(E)** oxidation-induced G>T transversions as an indicator of library preparation artifacts.
