# Supplementary material for: A Global Assessment of the Transcription-Dependent Single Nucleotide Variants Relies on the Characteristics of RNA-Sequencing Technologies
Source: Biomolecules. 2026 Jan 29;16(2):211. doi: 10.3390/biom16020211 (PMC12937670; doi:10.3390/biom16020211)
Supplement: Supplementary file 1 [file biomolecules-16-00211-s001.zip › Supplementary Parameters.pdf]

# File S1: Parameter settings of GATK and BCFtools

The parameter settings of GATK and BCFtools in pre-processing and calling are listed as follows:

For pre-process:

- SOAPnuke filter -l 10 -q 0.1 -n 0.01 -f AAGTCGGAGGCCAAGCGGTCTTAGGAAGACAA -r AAGTCGGATCGTAGCCATGTCGTTCTGTGAGCCAAGGAGTTG -1 read1.fastq.gz -2 read2.fastq.gz -o RNA -C read1.clean.fastq.gz -D read2.clean.fastq.gz
- STAR --runThreadN 20 --genomeDir STAR_hg38 --twopassMode Basic --readFilesIn read1.clean.fastq.gz read2.clean.fastq.gz --readFilesCommand zcat --quantMode TranscriptomeSAM GeneCounts --outFileNamePrefix RNA
- picard.jar SortSam INPUT=RNAAligned.out.sam OUTPUT=RNA.sort.bam SORT_ORDER=coordinate TMP_DIR=RNAtmp0
- java -jar picard.jar AddOrReplaceReadGroups PU=COMPLETE LB=COMPLETE RGPL=COMPLETE SM=RNA RGID=L0 VALIDATION_STRINGENCY=SILENT I=RNA.sort.bam O=RNA.sort.add.bam
- java -jar picard.jar MarkDuplicates REMOVE_DUPLICATES=true I=RNA.sort.add.bam M=RNA.rmdtxt O=RNA.sort.add.rmdup.bam VALIDATION_STRINGENCY=SILENT TMP_DIR=RNAtemp1
- java -jar picard.jar ReorderSam I=RNA.sort.add.rmdup.bam O=RNA.sort.add.rmdup.reorder.bam SD=hg38.fa TMP_DIR=RNAtemp2
- gatk SplitNCigarReads -R hg38.fa -I RNA.sort.add.rmdup.reorder.bam -O RNA.sort.add.rmdup.reorder.split.bam --tmp-dir RNAtmpdir

For GATK calling:

- gatk BaseRecalibrator -I RNA.sort.add.rmdup.reorder.split.bam -R hg38.fa --known-sites resources_broad_hg38_v0_Homo_sapiens_assembly38.dbsnp138.vcf -O RNArecalibration.table
- gatk ApplyBQSR -R hg38.fa -I RNA.sort.add.rmdup.reorder.split.bam --bqsr-recal-file RNArecalibration.table -O RNA.sort.add.rmdup.reorder.split.recall.bam
- gatk --java-options "-Xmx4g" HaplotypeCaller -R hg38.fa -I RNA.sort.add.rmdup.reorder.split.recall.bam -O RNA.sort.add.rmdup.reorder.split.recall.vcf.gz

- gatk VariantRecalibrator -R hg38.fa -V RNA.sort.add.rmdup.reorder.split.recall.vcf.gz --resource:hapmap,known=false,training=true,truth=true,prior=15.0 resources_broad_hg38_v0_hapmap_3.3.hg38.vcf --resource:omni,known=false,training=true,truth=false,prior=12.0 resources_broad_hg38_v0_1000G_omni2.5.hg38.vcf --resource:1000G,known=false,training=true,truth=false,prior=10.0 resources_broad_hg38_v0_1000G_phase1.snps.high_confidence.hg38.vcf --resource:dbsnp,known=true,training=false,truth=false,prior=2.0 resources_broad_hg38_v0_Homo_sapiens_assembly38.dbsnp138.vcf -tranche 100.0 -tranche 99.99 -tranche 99.98 -tranche 99.97 -tranche 99.96 -tranche 99.95 -tranche 99.94 -tranche 99.93 -tranche 99.92 -tranche 99.91 -tranche 99.9 -tranche 99.8 -tranche 99.7 -tranche 99.6 -tranche 99.5 -tranche 99.4 -tranche 99.3 -tranche 99.2 -tranche 99.1 -tranche 99.0 -tranche 98.0 -tranche 90.0 --max-gaussians 4 -an QD -an ReadPosRankSum -an FS -an SOR -mode SNP -O RNA.vcf.recal --tranches-file RNA.vcf.tranches --rscript-file RNA.vcf.plots.R
- gatk ApplyVQSR -R hg38.fa -V RNA.sort.add.rmdup.reorder.split.recall.vcf.gz --ts-filter-level 99.0 --tranches-file RNA.vcf.tranches --recal-file RNA.vcf.recal -mode SNP -O RNA.VQSR.vcf.gz
- gatk VariantFiltration -R hg38.fa -V RNA.VQSR.vcf.gz -O RNA.VQSR.filter.vcf.gz --filter-name "my_filter" --filter-expression "AB < 0.2 || MQ0 > 50"
- gatk SelectVariants --select-type-to-include SNP -V RNA.VQSR.filter.vcf.gz -O RNA.VQSR.filter.snp.vcf -R hg38.fa
- gatk SelectVariants -R hg38.fa -V RNA.VQSR.filter.snp.vcf -O RNA.VQSR.filter.snp.pass.vcf -select "vc.isNotFiltered()"

For BCFtools calling:

- bcftools mpileup -a AD -a DP -Ov -f hg38.fa RNA.sort.add.rmdup.reorder.split.bam | bcftools call -mv -Oz > RNA.bcftools.vcf.gz
- bcftools filter -Oz -o RNA.bcftools.filter.vcf.gz -s LOWQUAL -e 'QUAL<10 || FMT/DP <5' --SnpGap 5 --set-GTs . RNA.bcftools.vcf.gz
- bcftools view -Ov -v snps RNA.bcftools.filter.vcf.gz > RNA.bcftools.filter.snp.vcf
- grep -v '#' RNA.bcftools.filter.snp.vcf |awk -F '\t' '{if($7~/PASS/)print}' > RNA.bcftools.filter.snp.pass.vcf
